# Improving Information Technology Adoption and Implementation Through the Identification of Appropriate Benefits: Creating IMPROVE-IT

**DOI:** 10.2196/jmir.9.2.e9

**Published:** 2007-05-04

**Authors:** Kevin J Leonard, Dean F Sittig

**Affiliations:** ^2^Applied Research in Medical InformaticsNorthwest Permanente and Department of Medical Informatics and Clinical EpidemiologyOregon Health & Science UniversityPortlandORUSA; ^1^Department of Health PolicyManagement and EvaluationFaculty of MedicineUniversity of TorontoTorontoONCanada

**Keywords:** Information technology, IT cost, IT investment, Timing of benefits, Efficiencies, Cost-effectiveness, Health outcomes

## Abstract

This paper describes the objectives of a collaborative initiative that attempts to provide the evidence that increased information technology (IT) capabilities, availability, and use lead directly to improved clinical quality, safety, and effectiveness within the inpatient hospital setting. This collaborative network has defined specific measurement indicators in an attempt to examine the existence, timing, and level of improvements in health outcomes that can be derived from IT investment. These indicators are in three areas: (1) IT costs (which includes both initial and ongoing investment), (2) IT infusion (ie, system availability, adoption, and deployment), and (3) health performance (eg, clinical efficacy, efficiency, quality, and effectiveness). Herein, we outline the theoretical framework, the methodology employed to create the metrics, and the benefits that can be obtained.

## Introduction

A critical question is now facing health care: Does spending on information technology (IT) lead to greater system availability, increased clinician use, improved decision making, and better health outcomes? It is believed that one of the reasons health care systems have not widely adopted IT is that the benefits that emanate from investment in IT are poorly, if at all, defined [1-3]. In an attempt to address this need, this research initiative has developed several measures that link clinical system availability, use, and cost to clinical impact over a wide range of health care scenarios. Overall, it is the goal of IMPROVE-IT (indices measuring performance relating outcomes, value and expenditure through information technology) to demonstrate the relationship between IT and better health outcomes. Ultimately, the IMPROVE-IT project is attempting to provide a basis for the creation and dissemination of the evidence that increased IT capabilities, availability, and use lead directly to improved clinical quality, safety, and effectiveness, focusing primarily within the inpatient hospital setting [4].

The research literature has discussed the need for measuring the value associated with IT [5,6]. In order to accomplish this, there is a need to develop better methods for tracking IT spending, system availability, and utilization. Recent studies have attempted to estimate the business value generated from IT investment in health care in specific areas, but they have not recommended any method for measuring a broader (eg, hospital-wide) effect or for dealing with the problems of partial implementation [7-11]. In addition, studies are now underway to establish long-awaited quality benchmarks and performance measures for the health system across a number of perspectives, such as physician adherence to best practices [12], availability of systems [13], statistical modeling to predict efficiency [14], and overall scorecard development [15]. However, there has yet to be a detailed study examining the relationship between IT investment and improvement in financial efficiencies *and* health performance outcomes, where the results and benefits are observed.

It is hypothesized that IT spending provides an environment for a new and comprehensive level of care to exist. That is, without new technology and better information, clinicians would not be able to deliver the effective care that they can when these types of investment are made. IT can provide an opportunity to assess trends that formerly have taken much longer to identify. Improved information access can lead to rapid decision making relating to that information. Often, a clinical decision support tool is a component of the new system. Decisions aided by this support system may improve the operation of the organization: actions can now be taken sooner than they were historically, if they were taken at all. Finally, IT can be used to evaluate its own effectiveness by providing information on the improvement across a wide range of indicators. In short, better information can lead to better care, as demonstrated by improved health outcomes, such as creating the ability to diagnose patients more accurately, as well as sooner; to comply with patients’ wishes; to reduce the number or severity of errors; and to support care delivery through better access to information.

Although capturing and documenting IT investment, utilization, and outcomes may appear, on the surface, to be straightforward, there are many disparate factors and complexities that make this process extremely difficult. For example, while the concept of an electronic health record (EHR) seems clear, we believe that to actually create the infrastructure required to support EHRs, one must include many more aspects of clinical information and communications technologies. In an effort to simplify this process to some degree, in this paper we will use the term clinical information systems (CISs) to refer to this larger conglomeration of clinical information and communications technology. Secondly, before one can begin to manage such an effort, one must have the means (or availability) to measure that progress. Measuring the extent to which these systems have been deployed and are being used are the first two critical steps in measuring the overall impact these systems will ultimately have on the quality of care received by all patients.

As a result, and borrowing several concepts from conventional quality measurement efforts, it is clear that we must be able to measure aspects of the structure, process, and outcomes that make up these CISs. These concepts translate into measurements of health information management technology availability, use, and effectiveness at many different levels. For example, we believe that we must make these measurements at the single physician level, the clinic level, the hospital level, the entire health care organization level, and even the local, regional, and national levels. In addition, all of these measurements need to be made from the multiple viewpoints of the key users of these systems, namely, patients, clinicians, and those involved in population health activities (eg, public health departments). As in any large-scale measurement and evaluation effort, designing and validating the measures will be one of the most important and difficult challenges to overcome. In the immediate term, the objective is to entrench the philosophy of measurement through the selection of a “pilot” group of indicators being reported on in a hospital setting.

Going forward, contributing hospitals will be asked to provide measures on their hospital’s performance each quarter to a secured website. In exchange for this commitment, hospitals will be provided access to the secured website and all of the reported results (prior to publication). These results will be generated quarterly and will present performance measures and comparisons of individual member hospitals to an average “benchmark” as well as to other unidentified peer group hospitals.

## Phased Approach to Making Measurements

In addition to the conceptual model of the measurement system and identification of the key system users, we believe that we must use an iterative phased approach that will allow us to begin making measurements while we continue learning “how best to make these measurements.” This iterative approach will also allow us to move forward at varying rates in different organizations and even regions of the country. This is based on our firm belief that before one can expect to demonstrate improvements in any of the outcome measures associated with the CIS technology, we must first demonstrate that the key system users are actually using the information systems. Similarly, we believe that before we can expect to be able to measure any system use, we must be able to demonstrate that the requisite systems are in place and available to our key users. Therefore, we propose a three-phase iterative approach to beginning the measurements:

1. Phase I will consist of the measurements required to demonstrate “availability” of the systems.
2. Phase II will consist of the measurements required to demonstrate “use” of the systems.
3. Phase III will consist of the measurements required to demonstrate the effect of these systems on various performance measures that are often associated with IT use.

The first step in our research plan was to host a conference (November 11-12, 2004, Toronto, Canada) that would bring together people from a wide variety of stakeholder groups. In order to define the metrics, we needed to generate a consensus from many perspectives as to what was important to measure and how the measures should be calculated. On an ongoing basis, it is envisioned that these metrics would evolve and become much more comprehensive and complex; however, it is critical that the early-stage metrics be meaningful and feasibly generated from data that were clear, concise, and accessible.

The first day presentations included input from researchers, hospitals, integrated regions, consulting companies, vendors, and community care agencies. These presentations demonstrated first hand the strategy and the implementation of many information system initiatives throughout North America. The second day of the conference focused on measurement, highlighting the need to define a strategy and then implement a measurement and evaluation plan that reinforces that specific strategy. Then, post-implementation, the metrics can outline in detail both the successes and the failures. Finally, the conference ended with placing the participants into breakout groups to work on defining the indicators that we will begin to track. The goal of our conference was to arrive at a consensus regarding the origin of these indicators. These results from our conference are presented below.

## IT Costs

IT costs can be divided into four basic categories (adapted from van Bemmel and Musen [16]).

### Hardware

This is all of the equipment necessary for data input, processing, communication, and archiving (eg, personal computers, servers, routers, network cabling or wireless access points, and storage devices). One should also factor in the equipment necessary to insure system reliability, including battery backup systems, off-site data storage and fail-over systems, and even on-site emergency power generators. This equipment could be purchased, rented, or leased. These costs should include the initial purchase price, the expected amortization period (usually 3-5 years), depreciation, and maintenance and operating costs. (Operating expenses generally include items such as computer storage tapes and disks, paper, printer cartridges, and so on. While often considered a small part of the total IT costs, a general rule of thumb is that the yearly cost of a printer, including depreciation, maintenance, and operating expenses, is roughly equivalent to the original purchase price.)

### Software

This includes all of the software required to keep the organization functioning. This should include both system software such as the operating systems, database management systems, network operating systems, data communication software, and compilers (in the event that the organization is developing their own applications), along with the application software such as the results review, provider order entry, clinical documentation, admit/discharge/transfer, registration, scheduling, and billing. This software may be purchased, rented, or leased. These costs should include the initial purchase price or development costs, as well as ongoing maintenance contracts or costs (often one third of the original purchase or development costs).

### Personnel

This represents all of the people (both central, assuming a local hospital is part of a larger organization, and local) required to keep the systems working, including management, developers, implementers, technicians, and those charged with system and application maintenance. In addition, one should factor in the costs of the people charged with providing initial training (both the cost of the trainers as well as the time spent by the clinicians away from their jobs) and ongoing support to the clinicians (eg, help desk operators). An initial estimate of this number could be the number of full-time equivalents (FTEs) in the information technology department along with an average overhead cost associated with FTEs in your organization. (Overhead costs include such items as financial and personnel management, furniture, and telephone and mail services and are usually calculated as a percentage, roughly 10-40% of each individual’s salary and fringe benefit cost.)

### Space

This number should reflect the costs to purchase, maintain, and manage the space or real estate required to house all of the personnel and equipment associated with the IT department. In addition to the purchase price, rent, or leasing fees and their associated amortization and depreciation costs, one should also factor in the costs of providing heat, light, and cleaning services within these areas. An initial estimate of this number could be the total number of square meters taken up by the IT department. Clearly, the cost of this space will depend greatly on whether it is located within the hospital or at an off-site facility. It will also vary depending on the use of the space, for example, space for personnel probably costs significantly more than the space required to store backup disks or tapes.

Any specific measures developed or selected should be capable of taking into consideration at least the following three main methods an organization might use to obtain its IT solutions: (1) buy it from vendors, (2) build it themselves, (3) or outsource the work. In each of these three modes, one would expect that some of the cost categories would increase while others would decrease. For example, if you buy a system from a vendor, your software purchase costs should be higher, but your personnel software development costs would consequently be lower. Likewise, if an organization outsources their work, then you would expect to see significantly lower costs in all four of the IT investment categories, but one would have to add back in the cost of outsourcing the contract.

### IT Cost Measures

In order to begin this process of measurement, reporting, and analysis with as much consensus as possible, members of IMPROVE-IT convened to work on identifying the first generation metrics. One interesting debate focuses around whether the cost indicators should be just that, an indicator, or an all-inclusive cost calculation similar to a balance sheet item. In the end, the agreement was to focus on the former for two reasons. First, a simple straightforward indicator will be easier to calculate, which will entice more hospitals to submit their findings. Second, and perhaps more importantly, our emphasis is to identify a statistical relationship between IT spending and changes in health outcomes. As such, the actual amount invested is not as important as an *indicator* that can be considered as a predictor—not only of overall spending, but, hopefully, of changes in outcomes as well.

After much deliberation and consultation, the following measures were selected as the first generation of indicators along the cost axis:

- Amount of money spent on IT hardware over the last year: This straightforward indicator deals with current ongoing investment in hardware. It is hoped that this indicator will reflect the commitment to ongoing investment in new technology.
- Amount of money spent on software by the organization over the last year: This second investment indicator deals with the ongoing software costs.
- Total number of people on IT staff—FTEs: The third investment indicator will incorporate the human resources needed to operate and manage the new technology. Once again, this indicator will provide insight into the amount of support required.
- Amount of space: The final investment indicator simply measures the space required (ie, office space in square feet) to house the IT personnel and hardware for the organization.

## IT Infusion

IT availability can be defined as the existence of, and access to, the requisite technology to collect, store, display, and transmit patient-identifiable, structured, clinical data in electronic formats. Therefore, we must be able to identify whether health care institutions and their providers have access to various health IT components.

One such metric would be in the area of percentage of patients in a region who have their health data available in an electronic format. As measurement techniques become more sophisticated, the measure could be improved to estimate the “completeness” of each patient’s health record, although at the present time the definition of a “complete” EHR is still not precisely defined. On September 1, 2004, the American Health Information Management Association, Healthcare Information and Management Systems Society, and The National Alliance for Health Information Technology announced the formation of a Certification Commission for Healthcare Information Technology (CCHIT). They were charged with creating an efficient, impartial, and trusted mechanism for certifying ambulatory EHRs and other health care IT products. On January 7, 2007, the Commission announced that an additional set of 18 ambulatory EHRs had been certified, bringing the total to 55 [17].

Much of the current IT research literature, and practice, has focused on measuring and determining the optimal hardware and software configurations. What the industry truly needs, however, is analysis focused on the *use* of these computerized information systems and how they can provide organization-wide benefits.The adoption of new IT in the health care industry involves more than hardware and software issues. We need the ability to accurately measure the degree of “infusion” (or system capabilities), availability, and use of various CIS features so that we can begin comparing CIS implementations from different vendors at different organizations. While others [18] have developed very technically oriented measures, we believe [19] that one should go beyond technical attributes and focus on the behavior of clinicians to really answer the question, “What information technology is available and how is it used?”

This is not as straightforward a calculation as it might appear at first glance. Many subjective decisions are made independently by hospitals and other providers before any data are captured or analyses produced. These subjective decisions, which relate to what to capture, how to calculate it, and how to make the analysis relevant, all affect the final product. Due to the complexity of the concept of infusion, there are numerous options and metric calculations that can be selected. If two organizations make a different decision, which is almost a certainty, even if they happen to call the measure by the same name, the possibility is very low that they will compare identical factors. As a result, a cooperative venture is a necessary condition for meaningful comparisons. Once these measures have been agreed upon, then, and only then, can standards and baseline benchmarks be employed industry wide.

The general consensus is that *availability* and *use* of IT are two distinct concepts, and, therefore, we have identified three measures for each of these two separate concepts.

### IT Availability Measures

To measure availability, the first indicator is the number of clinical applications that are available to 50% or more of the clinicians in an organization. As a proxy, “available” is interpreted as clinicians who “have a login that allows them to access that part of the system.” Examples of the types of clinical applications we considered to be key components included:

- computer-based provider order entry (CPOE)
- computer-based order communication
- MD-level admitting, discharge, and daily progress notes
- RN-level nurse charting
- clinical laboratory results review
- picture archiving and communication systems (PACS)
- admit/discharge/transfer systems
- clinical data warehouse
- scheduling
- billing
- patient registration

Various types of clinical decision support (based on the Clinical Decision Support Implementers’ Workbook [20]) are available:

- proactive order sets
- preventive health maintenance reminders
- drug ordering alerts: drug-drug interactions, drug-allergy interactions, duplicate therapy
- access to online reference materials
- condition- or order-specific data displays
- support for complex clinical guidelines, protocols, or pathways

The second availability measure is the percentage of time the CIS was “available for use” by clinicians. We termed this as the percentage of system uptime. It should be calculated as follows:

% system uptime = 100 × (total time – scheduled downtime – unscheduled downtime) / total time

Where:

- Total time is the total number of minutes in a day times the number of days over which the measure is taken.
- Scheduled downtime includes all scheduled reasons for system unavailability, including system upgrades, routine hardware maintenance, system backups, etc.
- Unscheduled downtime includes all unscheduled reasons for system unavailability, including power outages, equipment failures, software lockups, etc.

The third availability measure is total number of unique patients with some type of clinical data available in the clinical repository. If possible, it would be better to factor in the number of years of data that each of these patients has available, which would allow us to calculate an availability measure of the total number of patient-years of clinical data available to clinicians. Another relatively simple proxy for this measurement could be the amount of disk space taken up by the clinical data contained in all the clinical systems. In the end, a simple count of unique patients was selected.

### IT Use Measures

IT use can be defined as actual hands-on employment of information systems by patients, providers, and those involved in population health. At the end-user level, this equates to actual use of various applications such as clinical results review or provider order entry. At the aggregate level, usage can be measured by the number of clinicians who routinely use the system to enter and review patient-level data.

The first CIS use measure is percentage of clinicians with an active user ID / password combination who actually log in to the system more than one time each day. We considered several other methods of “normalizing” the number of user log-ins, including number of log-ins per occupied bed and mean number of unique log-ins per individual patient. Once this measure reaches a uniformly high level, that is, the vast majority of institutions have well over 90% of their clinicians logging in each week, then we would consider revising this measure to reflect the mean percentage of all clinical applications available to each clinician that the clinician actually utilizes during the week or month.

For the second use measure, we selected percentage of patients with a completed chart (as defined by all needed data signed by all the appropriate clinicians) within 24 hours of their hospital discharge or outpatient visit.

For the final use measure, we focused on application-specific use measures. Here the goal would be to add one or more of these measures each year as our focus on the key clinical applications changes over time. Currently, there is tremendous emphasis on the use of computer-based provider order entry to reduce the number of errors in the ordering process; therefore, we chose the percentage of all orders entered directly by the person responsible for the patient’s care (who could be an MD, physician’s assistant, nurse practitioner, and the like) [21]. In subsequent years, we could easily imagine including, for example, the following:

- percentage of patients with a log-in to their personal health record who actually logged on in a given month
- percentage of clinicians who dictate their clinical notes (which currently requires the additional step of human transcription) rather than enter them directly via the keyboard
- overall percentage of clinical alerts or reminders that are overridden by clinicians

## Health Performance

State-of-the-art IT can potentially help clinicians and ancillary personnel to improve the overall care delivery process, which should lead to improvements in health outcomes [22]. These improvements will not occur unless there is a concerted effort to improve the process itself. Evaluating the impact of advanced IT on the health care delivery system requires not only standard measures, but the measurements must also demonstrate that the IT led to, or helped lead to, the observed clinical outcome. In other words, one must be able to infer a potential relationship between the use of the IT and the observed measure. Identifying these relationships can be difficult. In addition, our objective is to use, as much as possible, available health outcome or process measures that are already being used in other clinical quality, safety, and effectiveness evaluation practices (such as various Balanced Scorecard initiatives [23]). The following example measures attempt to document various aspects of the IT evaluation framework outlined above.

We have chosen the measures developed by the Center for Medicare and Medicaid Services of the United States as part of their National Voluntary Hospital Reporting Initiative to represent this area. While we recognized that these “process” measures do not represent actual health outcomes, we felt that in an effort to begin this work, it was most important to select measures that virtually all US hospitals were already making. In the first release of this measure, hospitals were asked to report their performance in three areas of care:

1. acute myocardial infarction (AMI): In the United States, approximately 1 million people suffer an AMI each year, making it one of the leading causes of hospital admission for patients age 65 and older.
2. congestive heart failure (CHF): CHF is the most common hospital admission diagnosis in patients aged 65 or older, accounting for more than 700000 hospitalizations across the United States each year.
3. community acquired pneumonia (CAP): This causes 4 million episodes of illness and nearly 1 million hospital admissions each year.

Within each of these focus areas, the Center for Medicare and Medicaid Services identified two to five specific measurements that hospitals were asked to report [24]. Therefore, our complete list of health outcome measures is as follows.

AMI:

- percentage of patients hospitalized with AMI who receive their initial treatment 30 min after arrival at the hospital
- percentage of patients who receive their percutaneous coronary intervention within 120 min of hospital arrival

CHF:

- percentage of patients hospitalized with CHF who receive their left ventricular assessment within 30 min after admission to the hospital
- percentage of patients hospitalized with CHF who were prescribed an angiotensin converting enzyme (ACE) inhibitor prior to discharge

CAP:

- percentage of patients hospitalized with CAP who receive their initial dose of antibiotics within 4 hours after admission to the hospital
- percentage of hospitalized pneumonia patients 65 years or older who were given a pneumococcal vaccine, if indicated, prior to discharge
- percentage of hospitalized pneumonia patients who have an arterial blood gas drawn, or who are monitored using pulse oximetry, within 24 hours of hospital arrival

Currently over 90% of the hospitals in the United States are reporting these measures. We anticipate being able to use the values reported through this voluntary reporting initiative as our proxy indicator of health outcomes for each hospital.

Finally, we realize that it is often difficult to identify a specific link or relationship between a specific CIS feature and many of these high-level process measures, but we cling to the belief that better, more accessible information, like that provided by a state-of-the-art CIS, should lead to better and more rapid clinical decision making. These improvements in decision making, or perhaps a simple reminder that a decision needs to be made, should in turn lead to improvements in these process measures.

## Measurement Going Forward

In order for the measurement to be meaningful, in addition to identifying the specific calculations, the size and type of hospital and the type of IT implemented must be consistent within peer groups. Therefore, each member hospital will need to be categorized on the following three factors:

1. number of beds
2. community care versus academic center
3. type(s) of IT

- physician/provider order entry (POE)
- electronic health record (EHR)
- clinical decision support (CDS)
- clinical data repository (CDR)
- ancillary systems interoperability (with internal systems such as labs, pharmacy, diagnostic imaging, emergency department triage systems)

This will result in much more meaningful comparisons as hospitals will be analyzed with respect to other similar, or peer group, hospitals.

Certainly, the specific outcome measures (either in terms of efficiency or effectiveness) that are calculated should relate in detail to the type of IT implemented (and being measured). The health outcome metrics presented above are intended to demonstrate the potential effect from any number of IT interventions. For example, the benefits for AMI patients could be the result of information delivery improvements related to the successful adoption of POE, EHR, CDS, CDR, or ancillary systems (such as emergency department IT). Other likely outcomes that must be further defined in order to relate to a designated IT system include the following:

- reduced length of stay
- lower readmission rates
- lower mortality
- reduced adverse events
- fewer complications with comorbidities
- faster turnaround cycle
- reduced human resource (doctors as well as other in-hospital staff) costs
- reduced diagnostic imaging costs
- reduced materials and supplies costs
- lower overall hospital costs (net of IT investment)

The following three steps emanate directly from the first generation metrics presented herein.

1. Establish national and international benchmarks for all common evaluation measures: Recruitment of membership is ongoing. We will use member data to develop a set of national and international cost, infusion, and effectiveness benchmarks. Benchmarks will be created to identify the 10th, 50th, and 90th percentiles of performance based on similar peer hospitals.
2. Explore statistical relationships between measures to illustrate potential cause and effect relationships: During the statistical analysis, we will identify the different factors that affect the timing and the amount of benefit that one should expect from IT investment, as this will allow for better prediction and easier management of expectations. Once a model of IT valuation is created, one of the primary benefits is the awareness of the interrelationships that exist among the many characteristics of the organization or its particular subindustry category.

3. Develop a complete and overall quality index that measures true impact of effective information systems in the inpatient setting: This will be accomplished over time as the data quality improves and the level of statistical analyses becomes more sophisticated.

## Conclusion

This research will study whether increased IT capabilities, availability, and use lead to improved clinical quality, safety, and effectiveness in the inpatient clinical setting. To reiterate, the logic underlying this hypothesis is as follows:

- Investment in IT inherently provides newer and more powerful technology and technological solutions.
- This improvement in “solution power” should then generate, and hence make available, “better” (more timely, valid, relevant, precise) information.
- Increasing the availability of this better information within the health care setting makes it more likely that decision makers will access or use this information to make better decisions.
- Finally, these better decisions should lead to results of increased efficiency (time and monetary gains) and effectiveness (improvement in measurable health outcomes across a variety of dimensions).

Consequently, it is anticipated that the mere act of identifying metrics, doing the calculations, and making the comparisons will have a positive impact on effective IT utilization in health care. There has been much established in the management literature pertaining to the act of measurement and the effect its mere presence can have on an outcome [25]. In particular, the Hawthorne Effect describes the fact that people perform better when they are being watched or measured, at least in the short term. The creation of indicators will highlight the importance of IT and will motivate the member hospitals to improve their results. Even if the measures identified herein are not optimal, they still serve a very important purpose of starting the debate as well as being the first steps in evaluating what is working and what is not.

In addition to the quantitative initiative presented here, we believe that similar qualitative studies should be conducted on the state of clinical and administrative information exchange standards and on the “values” of potential users of these systems. While these qualitative estimates of progress will not be as easy to interpret, they provide at least a glimpse of the progress that the industry is making in these critical arenas.

Examples of the types of topics these qualitative reviews might address include:

- qualitative assessment of the legal climate relating to public access to relevant data sources
- patient privacy protections
- legal restrictions on sending/receiving various data types
- electronic signatures
- prescription transmission to pharmacies
- legal restrictions on sending laboratory results to patients
- requirements to submit data to centralized databases
- availability of unique provider identification (UPI)

Likewise, in assessing the values of key system users, one might delve into:

- qualitative assessment of the perceived value of using IT for patient care
- incentives to adoption
- number of insurance companies reimbursing physicians for use of e-visits

Well-documented effects of health-related IT on health and health care represent vital metrics for the advancement of IT deployment. The value of the infrastructure ultimately must be evaluated, perhaps using the six quality attributes defined by the Institute of Medicine (safety, timeliness, efficiency, effectiveness, equitability, patient-centeredness) as measurement axes [26]. Although benefits and costs of IT have been measured in limited settings, measurements on the effects herein envisioned, on a national or international scale, have never been made. To accomplish this, of course, we must first establish the critical measurements of system availability and use.

In summary, while we firmly believe that the implementation and widespread adoption of IT throughout health care has had and will continue to have a significant positive effect, little documented evidence supports this belief. The IMPROVE-IT project is intended to demonstrate the tremendous positive influence that IT is having on health care. Improving efficiency requires knowledge of current inefficiencies, and improving effectiveness requires an understanding of the measurable outcomes of health care. No process can be managed or improved without first understanding the current status (ie, evaluating inherent performance measures).

## Multimedia Appendix 1

IMPROVE-IT Presentation, presented at Mednet 2006 (ppt)
					
										

## Multimedia Appendix 2

Quicktime Video Presentation, Kevin Leonard, Dec 2006
												
																	

## Abbreviations

AMI: acute myocardial infarction
CAP: community acquired pneumonia
CHF: congestive heart failure
CIS: clinical information system
EHR: electronic health record
IMPROVE-IT: indices measuring performance relating outcomes, value, and expenditure through information technology
IT: information technology
