# Supplementary material for: Improving Information Technology Adoption and Implementation Through the Identification of Appropriate Benefits: Creating IMPROVE-IT
Source: J Med Internet Res. 2007 May 4;9(2):e9. doi: 10.2196/jmir.9.2.e9 (PMC1874717; doi:10.2196/jmir.9.2.e9)
Supplement: Supplementary file 1 [file jmir_v9i2e9_app1.ppt]

## Slide 1
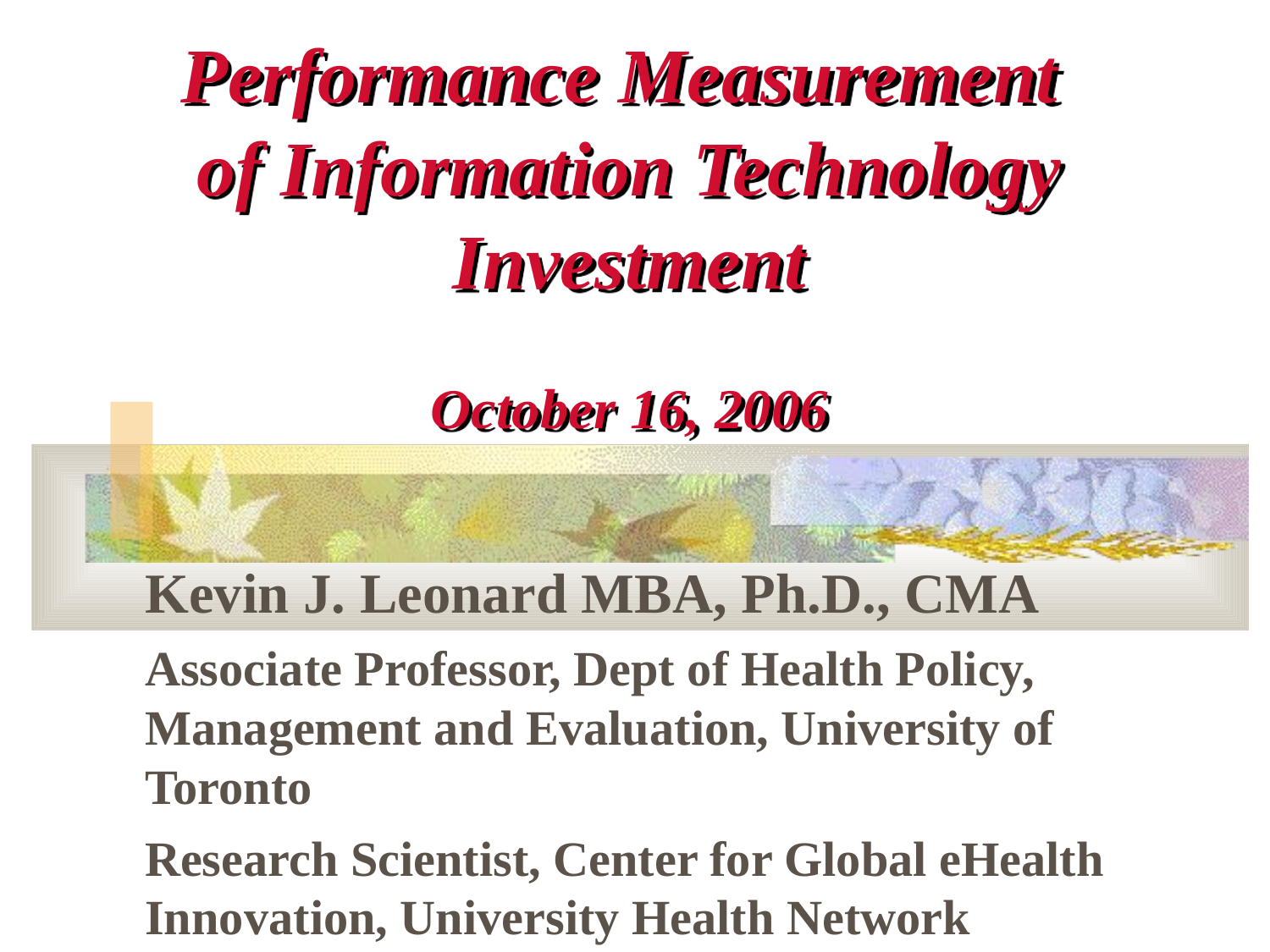

# Performance Measurement of Information Technology Investment October 16, 2006
Kevin J. Leonard MBA, Ph.D., CMA
Associate Professor, Dept of Health Policy, Management and Evaluation, University of Toronto
Research Scientist, Center for Global eHealth Innovation, University Health Network

## Slide 2
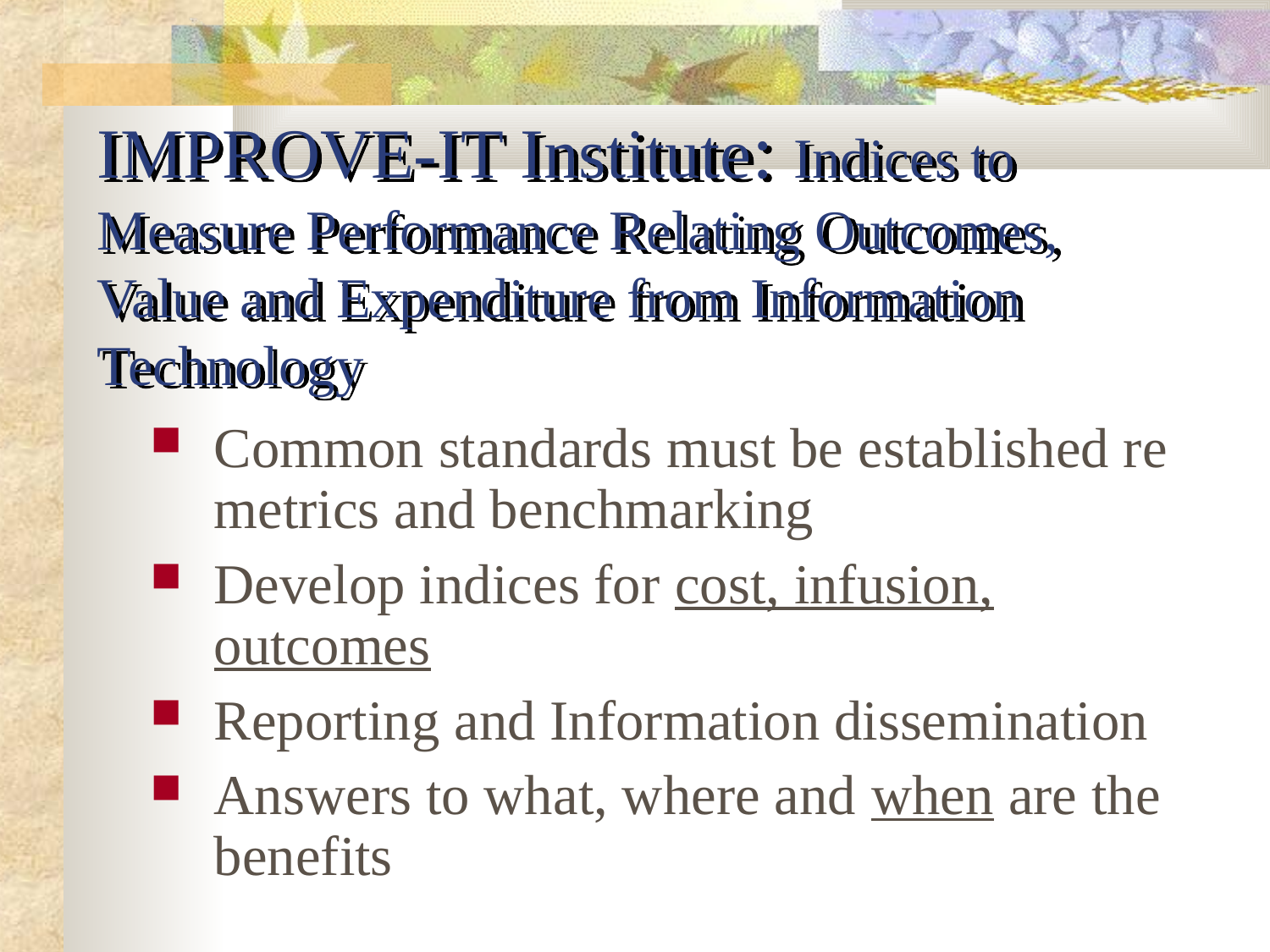

# IMPROVE-IT Institute: Indices to Measure Performance Relating Outcomes, Value and Expenditure from Information Technology
Common standards must be established re metrics and benchmarking
Develop indices for cost, infusion, outcomes
Reporting and Information dissemination
Answers to what, where and when are the benefits

## Slide 3
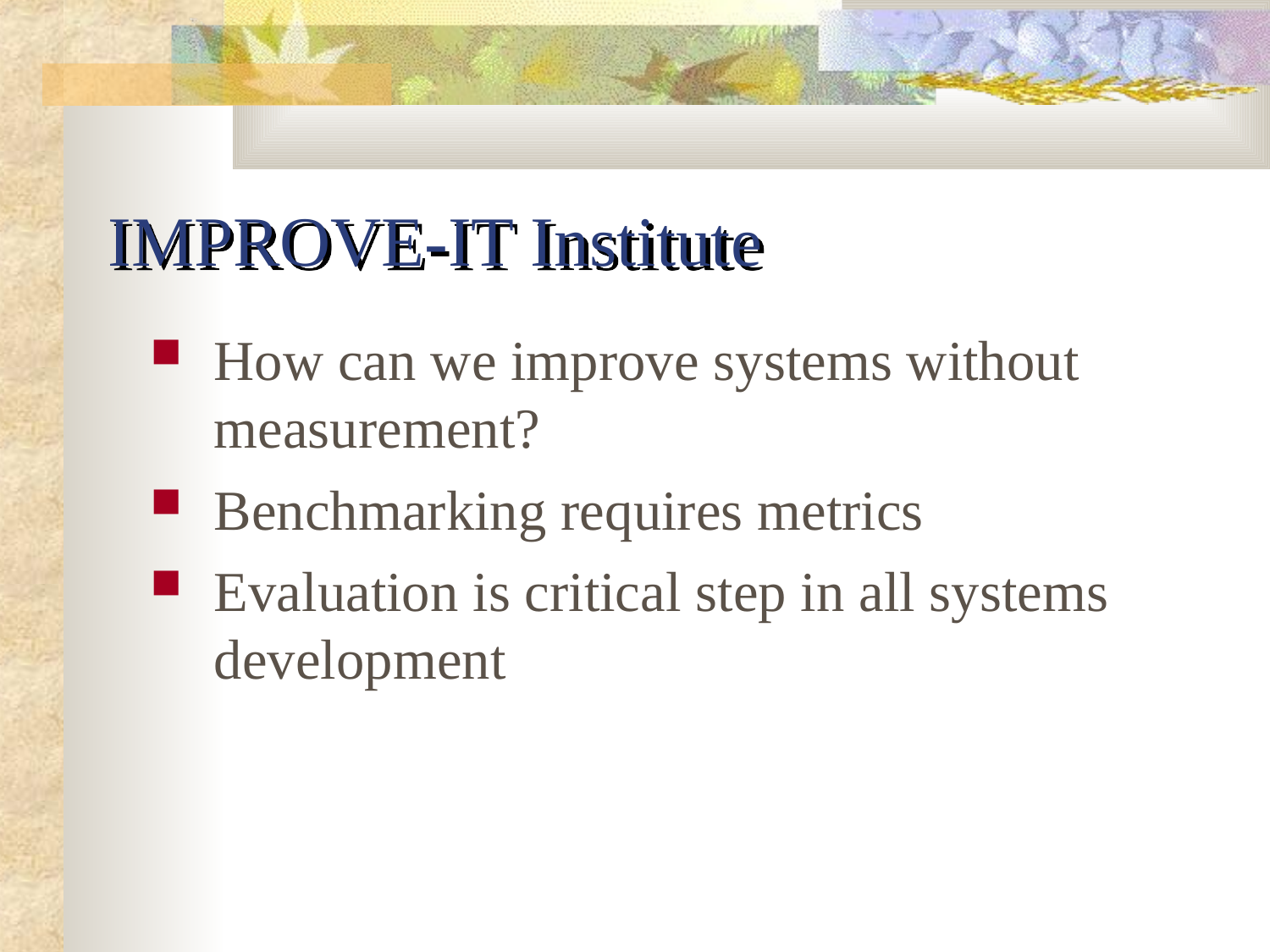

# IMPROVE-IT Institute
How can we improve systems without measurement?
Benchmarking requires metrics
Evaluation is critical step in all systems development

## Slide 4
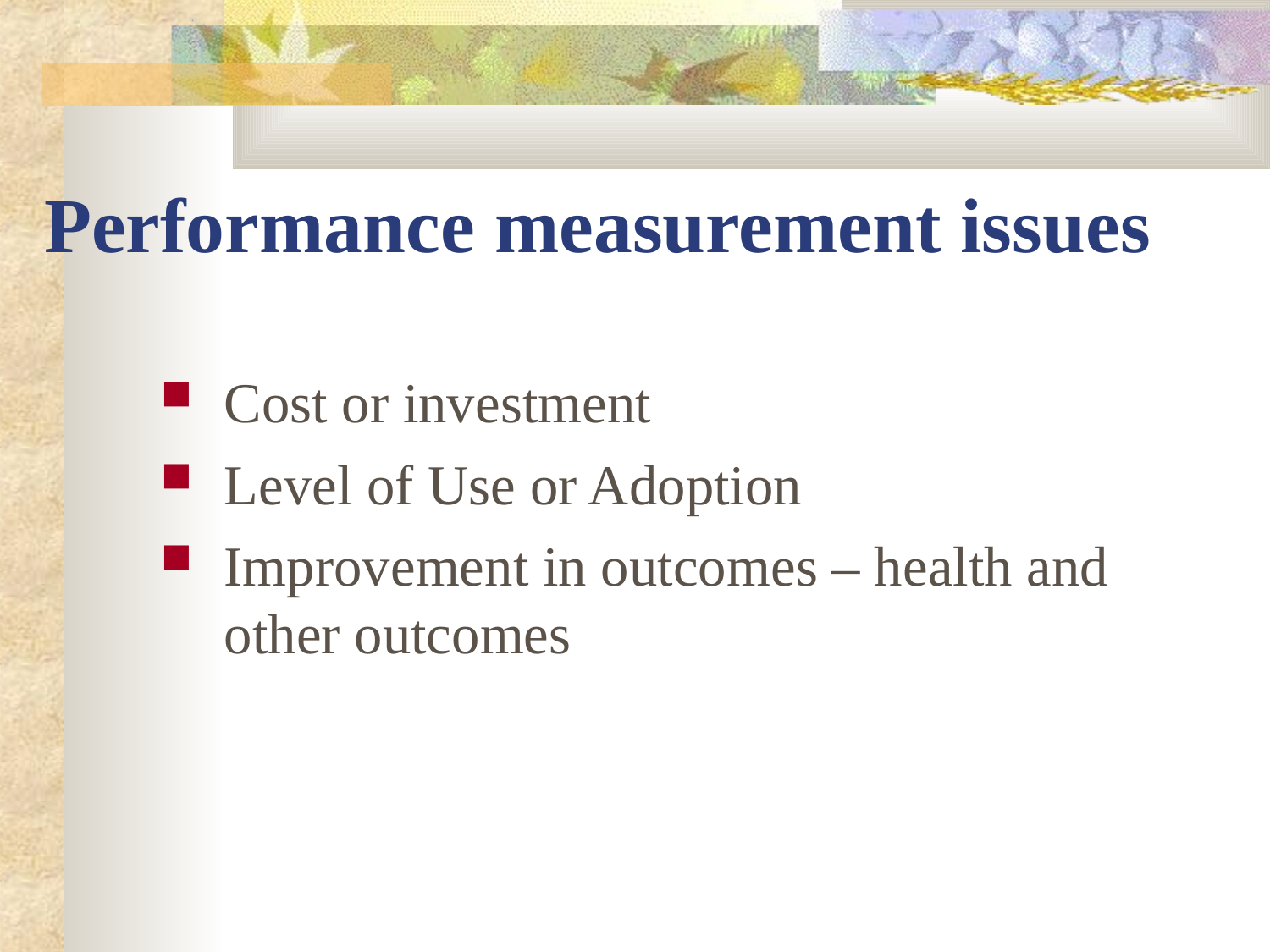

# Performance measurement issues
Cost or investment
Level of Use or Adoption
Improvement in outcomes – health and other outcomes

## Slide 5
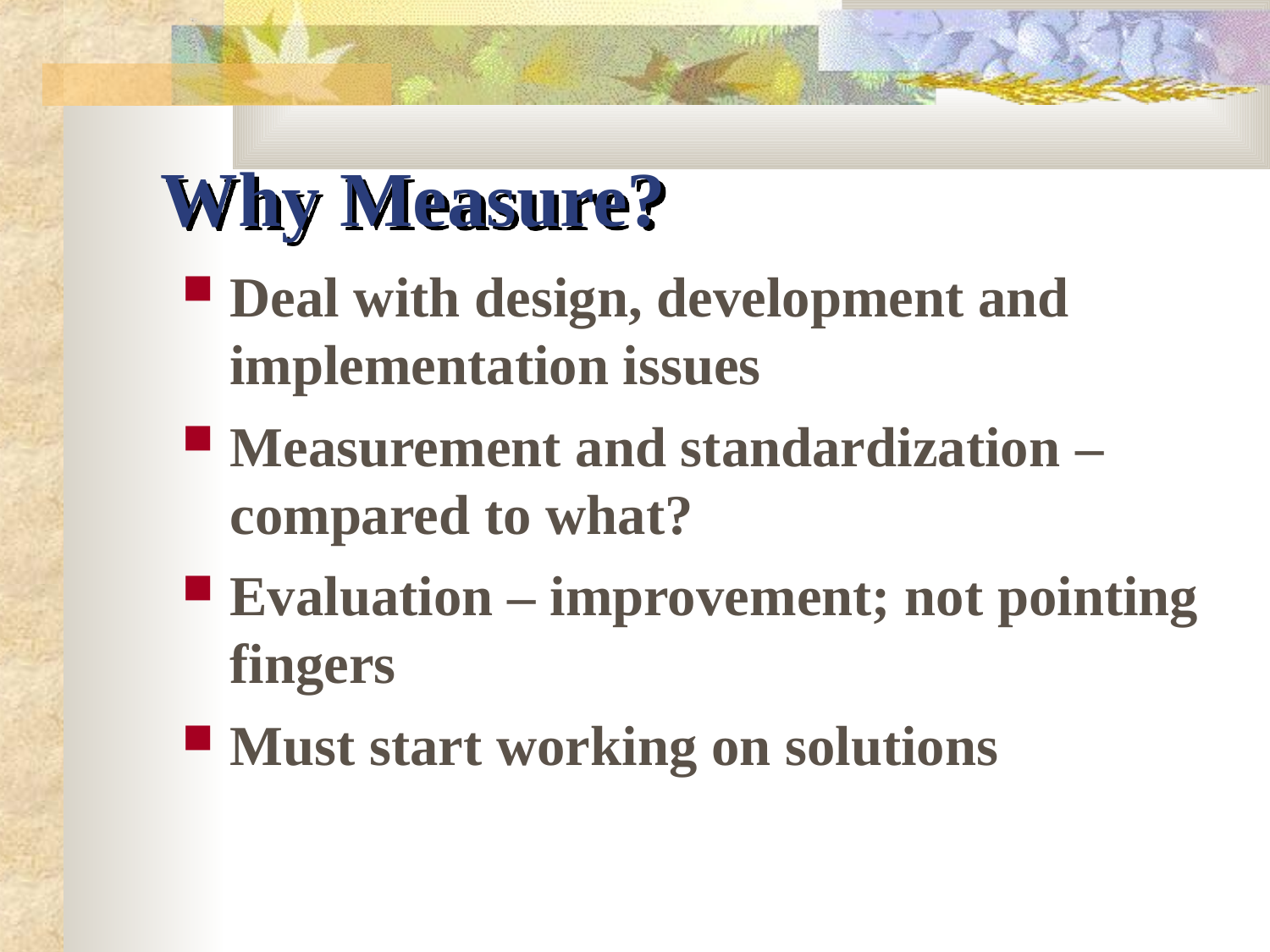

# Why Measure?
Deal with design, development and implementation issues
Measurement and standardization – compared to what?
Evaluation – improvement; not pointing fingers
Must start working on solutions

## Slide 6
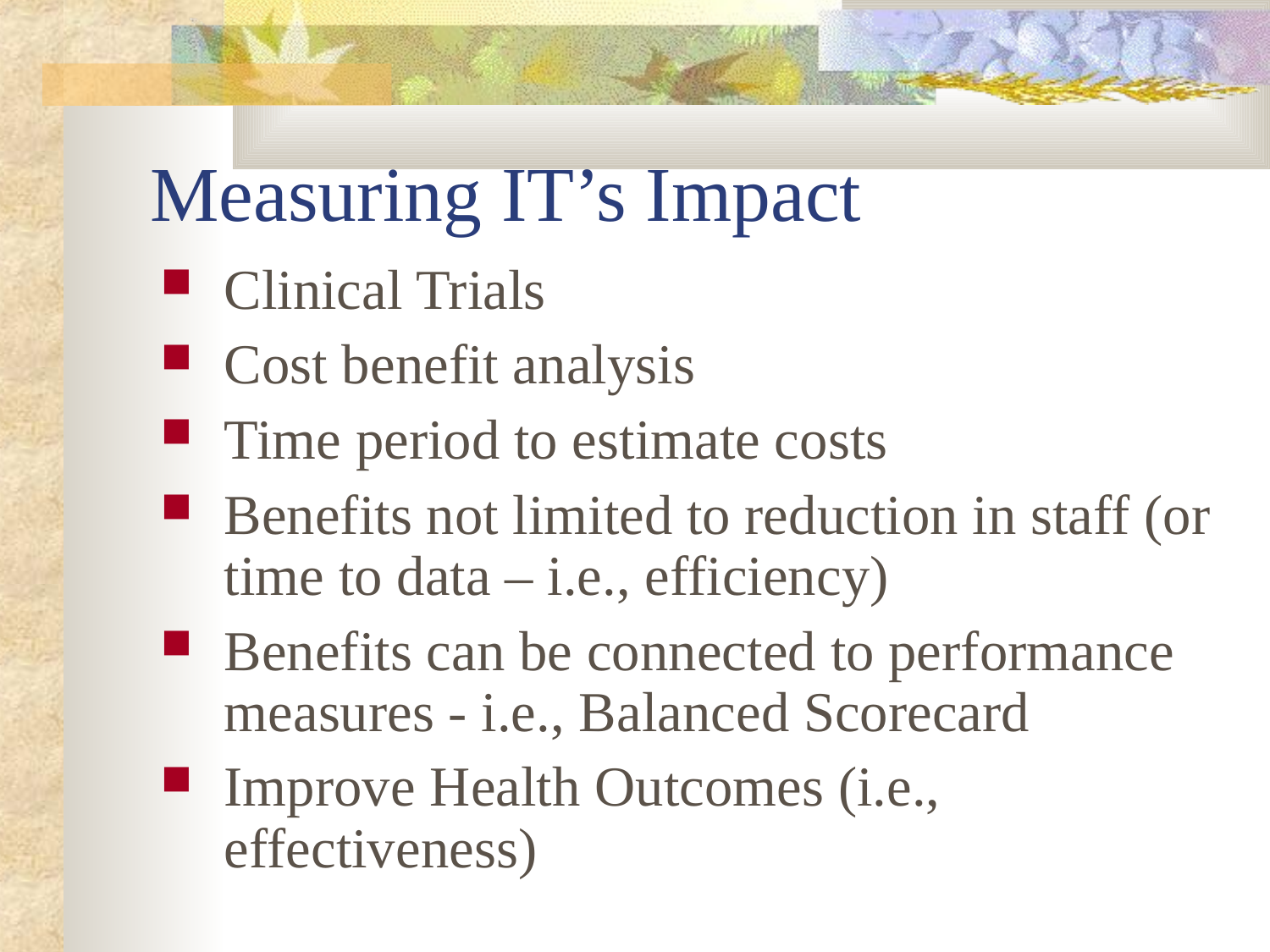

# Measuring IT’s Impact
Clinical Trials
Cost benefit analysis
Time period to estimate costs
Benefits not limited to reduction in staff (or time to data – i.e., efficiency)
Benefits can be connected to performance measures - i.e., Balanced Scorecard
Improve Health Outcomes (i.e., effectiveness)

## Slide 7
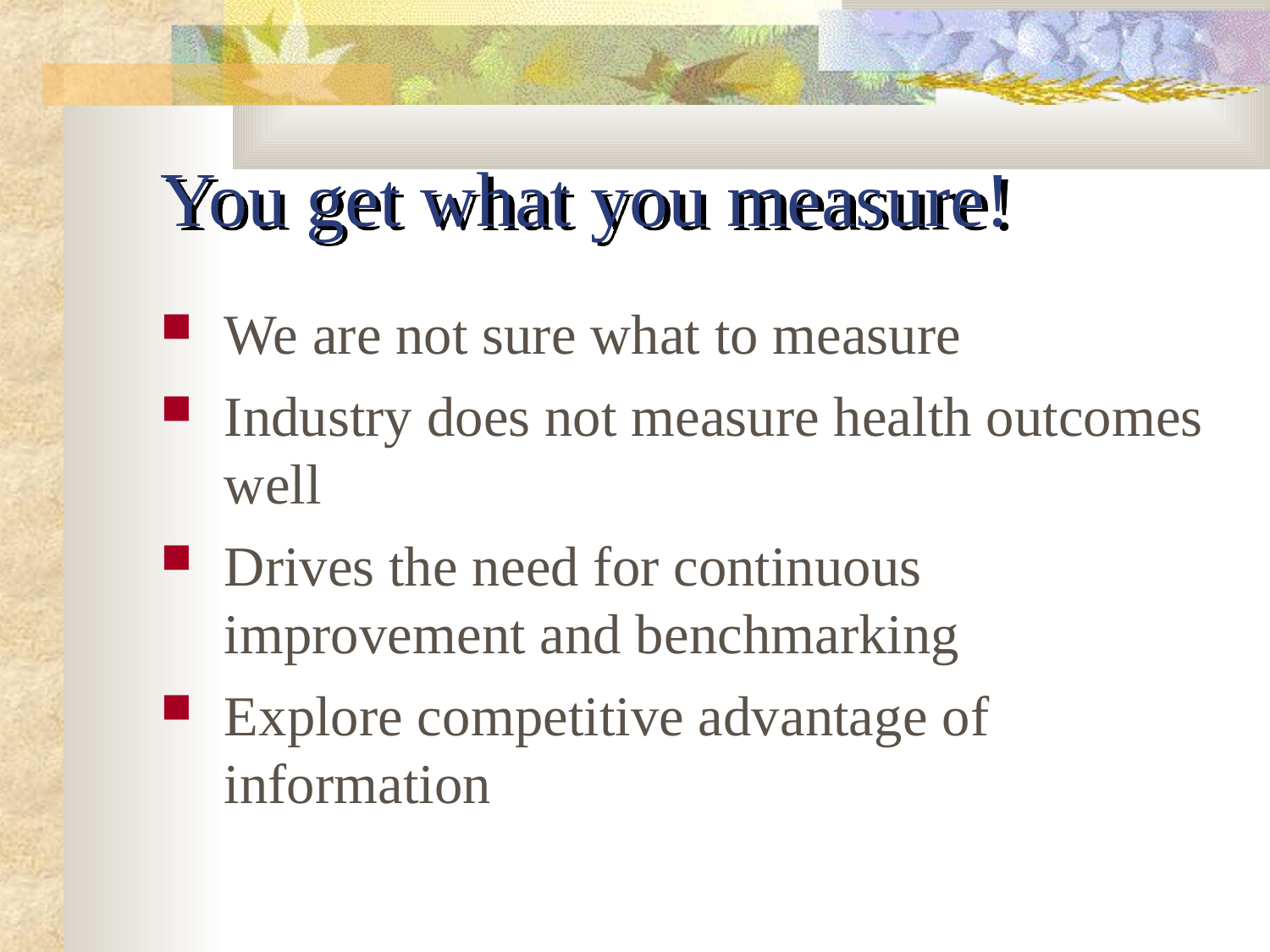

# You get what you measure!
We are not sure what to measure
Industry does not measure health outcomes well
Drives the need for continuous improvement and benchmarking
Explore competitive advantage of information

## Slide 8
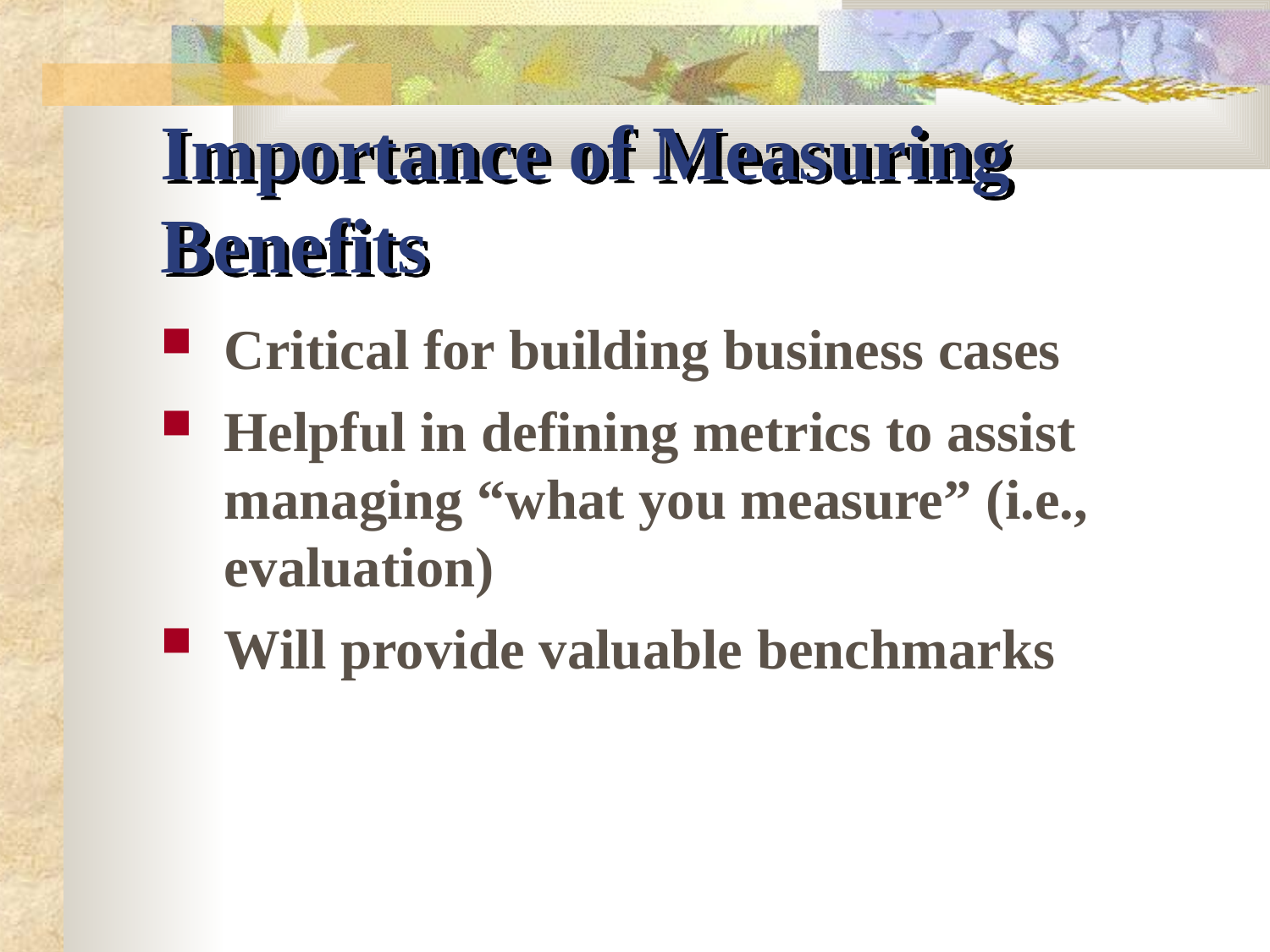

# Importance of Measuring Benefits
Critical for building business cases
Helpful in defining metrics to assist managing “what you measure” (i.e., evaluation)
Will provide valuable benchmarks
